# LPSF/GQ-02 Inhibits the Development of Hepatic Steatosis and Inflammation in a Mouse Model of Non-Alcoholic Fatty Liver Disease (NAFLD)

**DOI:** 10.1371/journal.pone.0123787

**Published:** 2015-04-14

**Authors:** Amanda Karolina Soares e Silva, Dilênia de Oliveira Cipriano Torres, Fabiana Oliveira dos Santos Gomes, Bruna dos Santos Silva, Edlene Lima Ribeiro, Amanda Costa Oliveira, Laise Aline Martins dos Santos, Maria do Carmo Alves de Lima, Ivan da Rocha Pitta, Christina Alves Peixoto

**Affiliations:** 1 Laboratório de Ultraestrutura, Centro de Pesquisa Aggeu Magalhães (FIOCRUZ), Pernambuco, Recife, Brazil; 2 Laboratório de Análises Clínicas da Universidade de Pernambuco, Pernambuco, Recife, Brazil; 3 Laboratório de planejamento e síntese de fármacos, Universidade federal de Pernambuco, Pernambuco, Recife, Brazil; University of Basque Country, SPAIN

## Abstract

Non-alcoholic fatty liver disease (NAFLD) defines a wide spectrum of liver diseases that extends from simple steatosis to non-alcoholic steatohepatitis. Although the pathogenesis of NAFLD remains undefined, it is recognized that insulin resistance is present in almost all patients who develop this disease. Thiazolidinediones (TZDs) act as an insulin sensitizer and have been used in the treatment of patients with type 2 diabetes and other insulin-resistant conditions, including NAFLD. Hence, therapy of NAFLD with insulin-sensitizing drugs should ideally improve the key hepatic histological changes, while also reducing cardiometabolic and cancer risks. Controversially, TZDs are associated with the development of cardiovascular events and liver problems. Therefore, there is a need for the development of new therapeutic strategies to improve liver function in patients with chronic liver diseases. The aim of the present study was to assess the therapeutic effects of LPSF/GQ-02 on the liver of LDLR-/- mice after a high-fat diet. Eighty male mice were divided into 4 groups and two different experiments: 1-received a standard diet; 2-fed with a high-fat diet (HFD); 3–HFD+pioglitazone; 4–HFD+LPSF/GQ-02. The experiments were conducted for 10 or 12 weeks and in the last two or four weeks respectively, the drugs were administered daily by gavage. The results obtained with an NAFLD murine model indicated that LPSF/GQ-02 was effective in improving the hepatic architecture, decreasing fat accumulation, reducing the amount of collagen, decreasing inflammation by reducing IL-6, iNOS, COX-2 and F4 / 80, and increasing the protein expression of IκBα, cytoplasmic NFκB-65, eNOS and IRS-1 in mice LDLR -/-. These results suggest a direct action by LPSF/GQ-02 on the factors that affect inflammation, insulin resistance and fat accumulation in the liver of these animals. Further studies are being conducted in our laboratory to investigate the possible mechanism of action of LPSF/GQ-02 on hepatic lipid metabolism.

## Introduction

Non-alcoholic fatty liver disease (NAFLD) is the most common cause of hepatic disease in western civilization [1] and is considered as a hepatic manifestation of a metabolic syndrome strongly associated with dyslipidemia, obesity, hypertension and insulin resistance[2]. NAFLD covers a spectrum of pathologies that range from simple hepatic steatosis to non-alcoholic steatohepatitis (NASH), which is characterized by cell ballooning, inflammation and different degrees of fibrosis [3]. Considering that the inflammation found in steatosis is usually benign, the most advanced state of NASH, and particularly fibrosing NASH, is one of the main causes of cirrhosis and mortality related to the liver [4].

One of the important and unresolved problems in NASH is the pathogenesis of hepatocyte injuries. One hypothesis for the pathogenesis of NAFLD is the “two-hit” hypothesis [5]. According to this paradigm, the primary abnormality (“first hit”) is most likely insulin resistance (IR), which leads to the accumulation of triglycerides within the hepatocytes. Then, a “second hit” induces the hepatocyte injury and inflammation (NASH) [6].

Hepatic inflammation can also induce insulin resistance via an imbalance in the secretion of pro-inflammatory cytokines, subsequent to activation of inflammatory/oxidative transcription factors [7]. A key transcription factor that mediates the inflammatory response in hepatocytes is nuclear factor κB (NF-B) [8, 9]. Activated hepatic NF-κB alone can drive insulin resistance, as evidenced by the finding that transgenic expression of the inhibitor of nuclear factor κB kinase subunit β (IKK-κβ), which increases NF-κB activity, resulted in overt insulin resistance in mice fed with a normal chow diet [10].

NF-κB activation increases the secretion of a number of pro-inflammatory cytokines, including interleukin (IL)-6, TNFα, and IL-1β [7]. NF-κB activation involves a complex series of signaling events that begins with the activation of the inhibitor κB (IκB) kinase complex, which, in turn, phosphorylates IκB [11, 12]. IκB is an inhibitor protein of NF-κB that binds to NF-κB, sequestering it in the cytoplasm [13]. However, once phosphorylated, IκB is targeted for ubiquitination and subsequent degradation, leaving NF-κB free to translocate to the nucleus and initiate the transcription of target genes [14]. These findings provide strong evidence that the liver is a primary site of the inflammatory action that causes insulin resistance, and that NF-κB is a central pathogenic factor underlying inflammation-induced insulin resistance.

Thiazolidinediones (TZDs) are a class of oral anti-diabetic medication that improves insulin resistance by acting as a selective agonist of the peroxisome proliferator activated receptor gamma (PPARγ) [15, 16]. Troglitazone, the first generation TZD, has been withdrawn from the market due to its hepatotoxicity [17], whereas rosiglitazone and pioglitazone are second generation TZDs and are currently available for clinical use [16, 18]. They redistribute fat from muscle and the liver to adipose tissue and thereby improve peripheral (skeletal muscle) and hepatic insulin sensitivity [16].

In general, TZDs improve hepatic histology in patients with NASH, although their favorable effect on steatosis is more striking than on other histological variables such as inflammation, ballooning or fibrosis. Their favorable effect on liver histology and liver biochemistry disappears upon their discontinuation, suggesting that long-term treatment is required to maintain their therapeutic benefits [19]. This is potentially a significant issue as recent studies have questioned the long-term safety of TZDs (especially rosiglitazone) [20]. Since the majority of the participants in these studies were non-diabetic, it is not clear if TZDs are equally effective in diabetics with NASH. Furthermore, it is possible that TZDs alone, without lifestyle modifications, may not be as effective [21].

Existing treatment for liver diseases is limited and differentiated, depending on the etiology and / or persistence of the stimulus. In general, current therapies try to stop or delay tissue injury, only managing to minimize damage in the cells in order to reduce the complications associated with the disease. When therapeutic measures are not effective, patients may progress to cirrhosis [22]. A liver transplant is the most effective treatment available for patients with chronic liver failure [23]. Therefore, there is a need for the development of new therapeutic strategies to improve the liver function of patients with chronic liver diseases.

In this context, our laboratory has already begun biological assessment studies with thiazolidine derivative LPSF/GQ-02 compound 5-(4-Chloro-benzylidene)-3-(4-methylbenzyl)-thiazolidine-2,4-dione, using biochemical, molecular, morphometric and ultra-structural analysis. The results have demonstrated that LPSF/GQ-02 was effective in decreasing the risk factors associated with the development of atherosclerosis, such as insulin resistance and inflammation, and consequently reducing atherosclerotic plaque in mice without the LDL receptor (LDLR-/-) [24]. Molecular modeling studies using nuclear PPAR-γ as molecular target available on the PDB database as 2PRG, suggested that LPSF/GQ-02 is a PPARγligand [25].

In the present study, mice with NAFLD, induced by a high-fat diet, were treated with LPSF/GQ-02 and assessed in relation to the development of hepatic steatosis and inflammation.

## Materials and Methods

### Ethics Statement

This study was carried out in accordance with the ethical principles in animal experimentation adopted by the Colégio Brasileiro de Experimentação Animal (COBEA). The protocol was approved by the Committee on the Ethics of Animal Experiments of the Fundação Oswaldo Cruz—FIOCRUZ (Permit Number: L-010/09).

### Synthesis of Thiazolidine Derivative LPSF/GQ-02

LPSF/GQ-02 representing the compounds 5-(4-chloro-benzylidene)-3-(4-methyl-benzyl)-thiazolidine- 2,4-dione was synthesized at the Department of Antibiotics of the Universidade Federal de Pernambuco (Brazil) following the methodology described by Mourão et al. [26].

### Study Design

Eighty mice were used, divided in 8 groups, all of which were homozygous for the absence of the LDL receptor gene (LDLR-/-), generated in the C57BL6/J background, obtained from Jackson Laboratories (USA) and bred in the vivarium of the Centro de Pesquisas Aggeu Magalhães. The state of the health of the mice was determined and they were acclimated in a laboratory environment with a temperature of 22°C (±1°) and artificial light from fluorescent lamps for a light/dark period of 12/12 hours. After weaning, the animals were submitted to a standard diet for eight days of adaptation. After this period, they received an atherogenic diet for 10 weeks [27] with drugs and they were separated into groups (n = 10) and submitted to daily treatment by gavage with pioglitazone and LPSF/GQ-02 for 15 days, as follows:

Control15—This group received a standard diet throughout the entire experiment.
HFD15—This group received the atherogenic diet (HFD) consisting of 21% milk fat and 1.25% cholesterol [27].
PIO15—This group received HFD and was treated with 20mg/kg/day of pioglitazone for 15 days [28].
LPSF/GQ-02–15—This group received HFD and was treated with 30mg/kg/day of glitazone LPSF/GQ-02 for 15 days [26].

A second experiment assessed the glitazones for a longer period of time. The animals were submitted to the abovementioned criteria, although they received the atherogenic diet for 12 weeks [29] and the drugs were administered during the last four weeks of the experimental diet, totaling 30 days of treatment with glitazones (Controle30/ HFD30/ PIO30/ LPSF/GQ-02–30). The atherogenic diet was acquired commercially (PragSoluções Biociências). The animals had free access to water and were kept in a controlled light cycle of 12 hours light/darkness. At the end of the therapy, the animals were anesthetized (Ketamine/Xylasine) before blood collection by cardiac puncture (without anticoagulant). The serum was separated and stored at -20°C for biochemical measurements. The aortas and livers were dissected and fixed for posterior processing (morphological analysis) and frozen at—80°C for posterior western blotting analysis.

### Biochemical Determinations

Serum levels of aspartate aminotransferase (AST), total cholesterol (TC), high-density lipoprotein (HDL), triglycerides (TGs), Low-density lipoprotein (LDL) and glucose were determined photometrically in the Cobas Integra 400 automatic analyzer (Roche, Mannheim, Germany), using Roche kits.

### Histopathology

Liver fragments were fixed in 10% formalin for 24 hours, before being processed and embedded in paraffin. Sections of 4–5μm were cut and mounted on glass slides. The sections were stained with hematoxylin-eosin (HE) and assessed with an inverted microscope (Observer Z1, Zeiss MicroImaging GmbH), equipped with a camera and 4.7.4 image analysis software (AxionCam MRm Zeiss), at a magnification of 400 x.

### Oil Red O Staining

In order to specifically detect lipids, samples of hepatic tissue were fixed in paraformaldehyde at 4% for 2h and embedded O.C.T (Tissue-Tek, Zoeterwoude, Netherlands) in the presence of liquid nitrogen. Afterwards, frozen cuts (8μm thickness) were made on a cryostat and the samples were then fixed with pure formaldehyde solution for 15 minutes. Next, the slides were stained with hematoxylin for 30 seconds to identify the nuclei of the cells. The cells were then washed in distilled water and stained with Oil Red O for 15 minutes. Five images of the same magnification were quantitatively analyzed using Gimp 2.6 software (GNU Image Manipulation Program, UNIX platforms).

### Determination of Liver Triglyceride Content

Livers were immediately collected and snap frozen in liquid nitrogen. A 50-mg piece of liver was homogenized in PBS. Folch’s reagent (CHCl3/MeOH, 2:1) (0.75 ml) was added to the homogenate. The nonaqueous phase was collected, and 30μl of 200 mg/ml Triton X-100 in CHCl3 was added. Samples were dried and triglycerides level was performed using the Roche kit by photometric method in the analyzer Cobas Integra 400, Roche, Mannheim, Germany.

### Picrosirius Red Staining

The cuts were stained with Sirius Red to assess the amount of collagen in the liver. The slides were pre-treated with xylene to remove paraffin and hydrated with a decreasing amount of ethanol. Subsequently, the cuts were stained with 1% Sirius Red solution in saturated picric acid for 2 hours and counter-stained with a solution of Fast green at 0.1% for 30 seconds. After this process, the slides were dehydrated in ethanol at 100%, cleared in xylene and mounted. Five images of the same magnification were quantitatively analyzed using Gimp 2.6 software (GNU Image Manipulation Program, UNIX platforms).

### Immunohistochemical Assays

Five sections (5 μm in thickness) of each group were cut and adhered to slides treated with 3-amino-propyl-trietoxi-silane (APES [Sigma, USA]). The sections were deparaffinized with xylene and rehydrated in graded ethanol (100 to 70%). To increase epitope exposure, the sections were heated for 30 minutes in a sodium citrate buffer (0.01 M, pH 6.0). To minimize endogenous peroxidase activity, the slides were treated with 0.3% (v/v) H_2_O_2_ in water for five minutes. The sections were washed with 0.01M PBS (pH 7.2) and then blocked with 1% BSA, 0.2% Tween 20 in PBS for 1h at room temperature. The sections were then incubated for 12 hours at 4°C with antibodies against IL-6 (1:100 eBioscience, San Diego, US), COX-2 (1:400 Abcam, Cambridge, UK), iNOS (1:100 Abcam, Cambridge, UK) and F4/80 (1:50 Abcam, Cambridge, UK). The antigen-antibody reaction was visualized with avidin-biotin peroxidase (Dako Universal LSAB + Kit, Peroxidase), using 3.3-diaminobenzidine as the chromogen. The slides were counterstained in hematoxylin. Positive staining resulted in a brown reaction product. Negative controls were treated as above, but with the omission of the first antibody. Five pictures at the same magnification were quantitatively analyzed using Gimp 2.6 software (GNU Image Manipulation Program, UNIX platforms).

### Cytosolic and Nuclear Protein Extraction

Cytosolic and nuclear proteins from the liver were isolated using Cayman’s Nuclear Extraction kit (Item No. 10009277, Cayman chemical company, Ann Arbor, Michigan, USA). The liver fragments were homogenized in a hypotonic buffer supplemented with DTT and Nonidet P-40 per gram of tissue. The livers were centrifuged and re-suspended by adding specified assay reagents, following the manufacturer’s instructions. The cytosolic and nuclear fractions were stored in pre-chilled vials at -80°C until further analysis. Liver cytosols were used to determine the quantity of IκBα, IRS-1, CD-220, ABCA1, eNOS and NFκB p65 in the immunoblotting, whereas nuclear fractions were used for PPARα and PPARγ immunoblotting.

### Measurement of Protein Levels

The total, cytosolic and nuclear extraction protein levels were determined using the Bradford method, with bovine serum albumin as standard [30]. The samples were read in a spectrophotometer at 660nm. All samples were run in duplicate and the mean of the two absorbency levels was used to determine the protein quantity. The protein concentration per sample amount was determined using the equation from a calibration curve. The curve was generated using the same method as the samples, with the substitution of bovine serum albumin at five concentration levels.

### Western Blot Analysis

The proteins (40 μg) were separated to 12% (NF-κB p65, IκBα, ABCA1, eNOS, PPARα, PPARγ, IRS-1 and CD-220) sodium dodecyl sulfate–polyacrylamide by gel electrophoresis under reduced conditions and were electrophoretically transferred onto a nitrocellulose membranes (Bio Rad, CA, USA, Ref. 162–0115). After overnight blocking at 4°C with 5% non-fat milk in TBS-T (Tris-buffered saline 0.1% plus 0.05% Tween 20, pH 7.4), the membranes were incubated at room temperature for 3h with antibodies against the following: NF-κB p65 (1:200, Santa Cruz Biotechnology, CA); IκBα (1:500, Santa Cruz Biotechnology, CA); ABCA1 (1:1000, Abcam Cambridge, UK); eNOS (1:1000, Abcam Cambridge, UK); PPARα (1:1000, Abcam Cambridge, UK); PPARγ (1:1000, Abcam Cambridge, UK); IRS-1 (1:1000, Abcam Cambridge, UK) and CD-220 (1:1000, Abcam Cambridge, UK), diluted in TBS-T buffer solution containing 3% non-fat milk. After washing (six times, 10 min each) in TBS-T, the membranes were further reacted with horseradish peroxidase-conjugated anti-rabbit antibody (1:80000, Sigma, USA), diluted in TBS-T with 1% nonfat milk, for 1h 30min at room temperature. An enhanced chemiluminescence reagent (Super Signal, Pierce, Ref. 34080) was used to visualize the labeled protein bands and the blots were developed on X-ray film (Fuji Medical, Kodak, Ref. Z358487-50EA). For quantification, the density of pixels of each band was determined by Image J 1.38 software (available at http://rsbweb.nih.gov/ij/download.html; developed by Wayne Rasband, NIH, Bethesda, MD). The results were confirmed in three sets of experiments for each protein investigated. Immunoblotting for β-actin was performed as a control for the above protein blots. After protein blot visualization with enhanced chemiluminescence, the protein antibodies were stripped from the membranes, which were reprobed with monoclonal anti-β-actin antibody (1:2000, Sigma, USA), and protein densitometry was performed.

### Statistical Analysis

GraphPad Prism software (version 5) was used for the statistical analysis. Data were expressed as mean ± standard deviation. Differences between the control and treated groups were analyzed using analysis of variance (ANOVA), prior to the performance of Tukey’s post hoc test or the Student's t-test. Probability values less than 0.05 were considered significant.

## Results

### Biochemical Determinations

**Fig 1:**
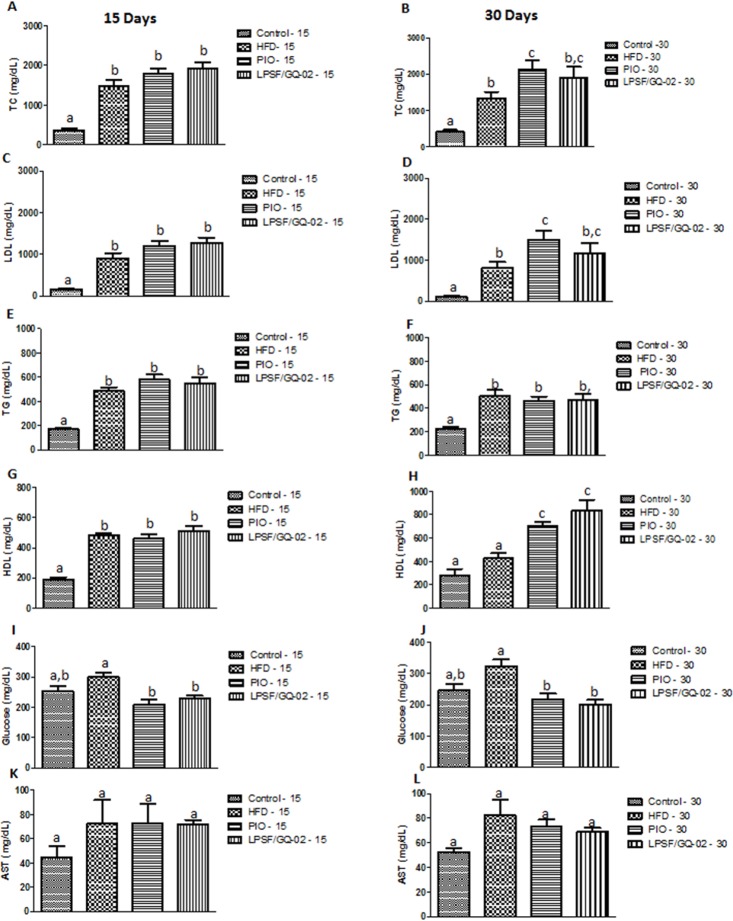
Effect of treatment with Pioglitazone and LPSF/GQ-02 for 15 and 30 days on biochemical blood parameters in LDLR−/− mice. TC—total cholesterol, LDL—low-density lipoprotein cholesterol, TG—triglycerides, HDL—high-density lipoprotein cholesterol, AST—aspartate aminotransferase. Values were determined in plasma samples from nonfasting animals. Values expressed as mean ±S.D. Biochemical determinations performed with eight animals per group. Different letters denote significant differences between treatments, P< 0.05.

After 15 and 30 days, the high-fat diet group showed a significant increase in total and LDL cholesterol and triglycerides compared to the control group. Similarly, treatment pioglitazone and LPSF/GQ-02 did not reduce total and LDL cholesterol and triglycerides levels (Fig 1A–1F), even treatment with pioglitazone for 30 days significantly increased levels of total and LDL cholesterol compared with HFD group (Fig 1D)

Treatment with pioglitazone and LPSF/GQ-02 did not alter the HDL levels compared with HFD group after 15 days (Fig 1G). However, after 30 days of treatment, pioglitazone and LPSF/GQ-02 induced a significant increase in serum HDL, indicating a time-dependent effect (Fig 1H). Serum glucose levels were evaluated after 15 and 30 days of treatment. There was a significant decrease in serum glucose levels after the use of pioglitazone and LPSF/GQ-02 compared with the HFD group (Fig 1I and 1J). Finally, in relation to the serum AST, no significant difference was observed between groups (Fig 1K and 1L).

### Effects of LPSF/GQ-02 on the Hepatic Architecture

**Fig 2:**
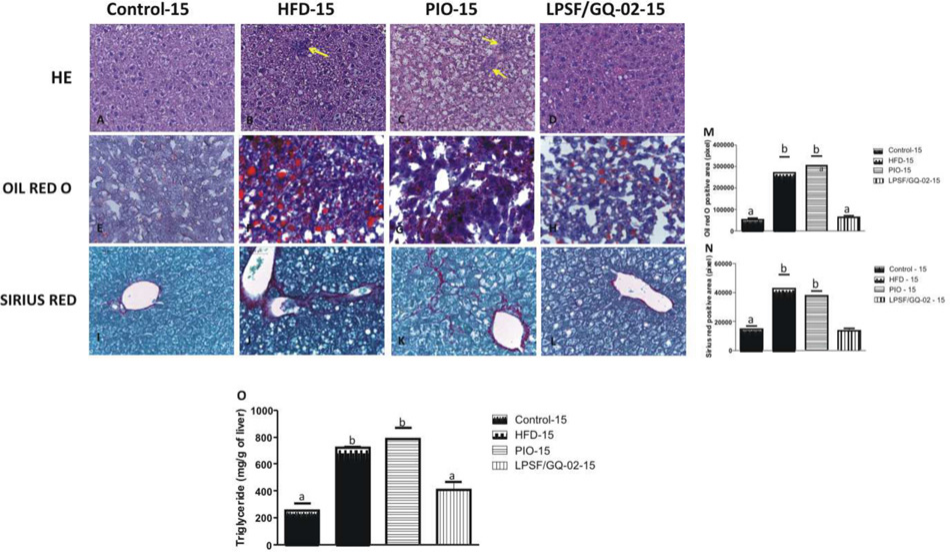
Effects of Pioglitazone and LPSF/GQ-02 for 15 days- hepatic histopathological analyses of LDLR-/- mice. Representative liver sections stained with H&E (A–D), oil red O (E–H) and Sirius Red (I-L): (A) Control group. (B) HFD group exhibiting macro and microvesicular steatosis and inflammatory infiltrates (arrow). (C) Pioglitazone group showing steatosis and inflammatory infiltrates (arrow). (D) LPSF/GQ-02 group showing normal hepatic architecture tissue organization, with few lipid droplets. (E) Control group. (F) HFD group with increase in hepatic lipids. (G) Pioglitazone group showing increase in lipids content (H) LPSF/GQ-02 presenting few lipid inclusions. (I) Control group. (J) HFD group showing a great quantity of collagen in the perivenular and sinusoidal regions. (K) Pioglitazone group exhibiting a great quantity of collagen. (L) LPSF/GQ-02 group exhibiting collagen only in the perivenular region. Bars 20μm. (M,N) Quantification of labelling for Oil Red O and collagen by Sirius Red (N = 5), respectively. (O) Quantification of liver triglyceride (N = 5). Different letters denote significant differences between treatments, P< 0.001 and P< 0.0001, respectively.

Histological analysis of the hepatic fragments in the control15 group exhibited well-preserved architecture with characteristic hepatocytes distributed homogenously throughout the hepatic parenchyma (Fig 2A). After induction to the high-fat diet, the HFD15 group exhibited significant alterations, including tissue disorganization with macro and microvesicular steatosis in the cytoplasm of the hepatocytes, as well as the presence of multiple foci of inflammable infiltrates (Fig 2B). The pioglitazone group was not capable of reversing the alterations caused by the high-fat diet and exhibited severe tissue disorganization, macro and microvesicular steatosis and many inflammatory infiltrates throughout the hepatic tissue (Fig 2C). The group that received the diet and was treated with LPSF/GQ-02 for 15 days showed a significant improvement in the hepatic architecture, with steatosis reduction as well as inflammation, which was confirmed by the reduction of foci of inflammatory infiltrates (Fig 2D).

**Fig 3:**
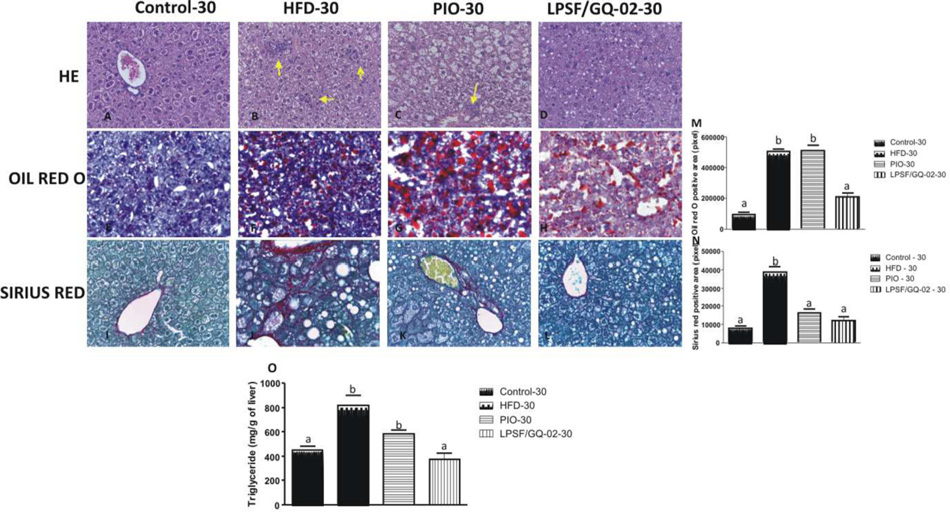
Effects of Pioglitazone and LPSF/GQ-02 for 30 days- hepatic histopathological analyses of LDLR-/- mice. Representative liver sections stained with H&E (A–D), oil red O (E–H) and Sirius Red (I-L): (A) Control group. (B) HFD group exhibiting macro and microvesicular steatosis and inflammatory infiltrates (arrow). (C) Pioglitazone group showing steatosis and inflammatory infiltrates (arrow). (D) LPSF/GQ-02 group showing normal hepatic architecture tissue organization, with few lipid droplets. (E) Control group. (F) HFD group with increase in hepatic lipids. (G) Pioglitazone group showing increase in lipids content (H) LPSF/GQ-02 presenting few lipid inclusions. (I) Control group. (J) HFD group showing a great quantity of collagen in the perivenular and sinusoidal regions. (K) Pioglitazone group exhibiting a few quantity of collagen. (L) LPSF/GQ-02 group exhibiting collagen only in the perivenular region. Bars 20μm. (M,N) Quantification of labelling for Oil Red O and collagen by Sirius Red (N = 5), respectively. (O) Quantification of liver triglyceride (N = 5). Different letters denote significant differences between treatments, P< 0.001 and P< 0.0001, respectively.

A second experiment, involving the administration of drugs for 30 days, assessed the time-dependant action of both pioglitazone and LPSF/GQ-02. The histological analysis revealed patterns similar to the groups that received the drug for only 15 days. The control group maintained the same characteristics (Fig 3A). However, the HFD30 and PIO30 groups exhibited a greater severity in the accumulation of lipids in the cytoplasm of hepatocytes and increased inflammation, confirmed by the greater presence of inflammatory infiltrates (Fig 3B and 3C). The group that received LPSF/GQ-02 reduced macro and microvesicular steatosis and decreased the quantity of inflammatory infiltrates, reestablishing a hepatic architecture similar to that found in the control group (Fig 3D).

### LPSF/GQ-02 Decreased the Levels of Hepatic Fat After Induction of a High Fat Diet

The accumulation of fat in the liver is the key point in the appearance of NAFLD. Consequently, the quantity of lipids in the liver was analyzed using the specific staining of Oil red O. The control groups from both the 15-day and 30-day experiments exhibited a basal amount of lipids, characterizing a normal pattern (Figs 2A and 3A). After the induction of the high-fat diet, there was a significant increase in the quantity of lipids, when compared to the control group, in both the 15-day and 30-day experiments (Figs 2B and 3B). The pioglitazone groups (15 and 30 days) did not decrease the quantity of fat in the liver when compared to the HFD group (Figs 2C and 3C). However, the use of LPSF/GQ-02 brought about a significant decrease in the quantity of fat in the hepatic tissue when compared to the HFD and pioglitazone groups (Figs 2D and 3D). Quantitative analysis was performed using ANOVA and Tukey’s post hoc tests (Figs 2M and 3M).

Additionally, liver triglyceride content was determined. The results obtained confirmed the specific staining of Oil red O analyses. Liver triglyceride content was significantly elevated after the high fat diet, and pioglitazone treatment had no effect on liver triglycerides On the other hand, the LPSF/GQ-02 promoted a significant decrease compared to HFD groups and pioglitazone (Figs 2O and 3O).

### Action of LPSF/GQ-02 on Hepatic Fibrosis

Sirius Red staining was used to analyze the evolution of fibrosis since it interacts strongly with the basic amino acids of molecules from different types of collagen [31]. Both the 15-day and 30-day control groups exhibited normal patterns, with collagen deposition in the perivenular region (Figs 2A and 3A). The HFD groups exhibited significant collagen accumulation when compared to the control group. There was an increase in the amount of collagen in the perivenular and sinusoidal regions, especially in the 30-day group (Figs 2B and 3B). The group treated with pioglitazone for 15 days was not effective in decreasing the collagen content when compared with the HFD group, exhibiting the same collagen deposition pattern. However, in the group treated with pioglitazone for 30 days, there was a reduction in collagen when compared with the HFD group (Figs 2C and 3C), indicating that the action of the drug, in terms of decreasing fibrosis, occurs in a time-dependant manner. LPSF/GQ-02 significantly reduced collagen in the 15-day and 30-day groups when compared with the HFD and pioglitazone groups, exhibiting a pattern similar to the control group, with collagen only found in the perivenular region ((Figs 2D and 3D). Quantitative analysis of the collagen content was performed in all groups using ANOVA and Tukey’s post hoc tests (Figs 2N and 3N).

### LPSF/GQ-02 Decreased the Levels of Inflammatory Markers in Mice with NAFLD

**Fig 4:**
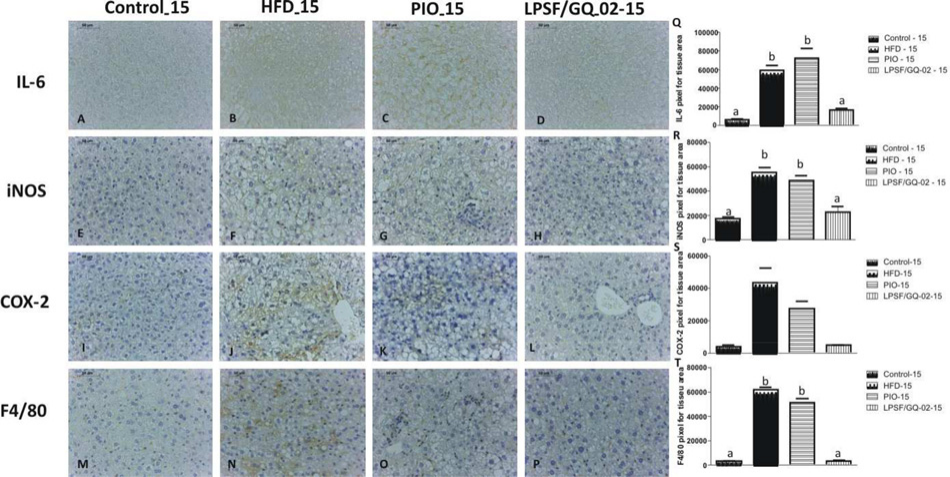
Immunohistochemical analysis for interleukin 6 (IL-6), inducible nitric oxide synthase (iNOS), cyclooxygenase 2 (COX-2) and F4/80 in LDLR-/- mice treated with Pioglitazone and LPSF/GQ-02 for 15 days. (A, E, I, M) Control group exhibited low reactivity to IL-6, iNOS, COX-2 and F4/80. HFD group showed high reactivity (B, F, J, N). Similarly, Pioglitazone group exhibited strong reaction for IL-6, iNOS, COX-2 and F4/80 (C, G, K, O). (D, H, L, P). Conversely, LPSF/GQ-02 groups exhibited reduced reactivity for IL-6, iNOS, COX-2 and F4/80. Bars 50μm. (Q,R,S,T) Quantification of labeling for IL-6, iNOS, COX-2 and F4/80 (N = 5). Different letters denote significant differences between treatments, P< 0.0001, P< 0.005, P< 0.005, P< 0.0001 respectively.

**Fig 5:**
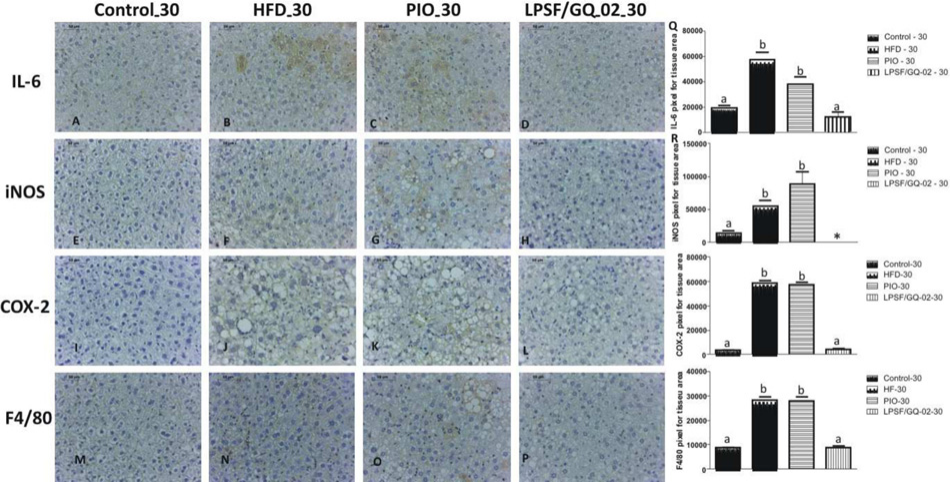
Immunohistochemical analysis for interleukin 6 (IL-6), inducible nitric oxide synthase (iNOS), cyclooxygenase 2 (COX-2) and F4/80 in LDLR-/- mice treated with Pioglitazone and LPSF/GQ-02 for 30 days. (A, E, I, M) Control group exhibited low reactivity to IL-6, iNOS, COX-2 and F4/80. HFD group showed high reactivity (B, F, J, N). Similarly, Pioglitazone group exhibited strong reaction for IL-6, iNOS, COX-2 and F4/80 (C, G, K, O). (D, H, L, P). Conversely, LPSF/GQ-02 groups exhibited reduced reactivity for IL-6, iNOS, COX-2 and F4/80. Bars 50μm. (Q,R,S,T) Quantification of labeling for IL-6, iNOS, COX-2 and F4/80 (N = 5). Different letters denote significant differences between treatments, P< 0.0001, P< 0.005, P< 0.005, P< 0.0001 respectively.

IL-6 is a multifunctional cytokine that regulates immune responses, as well as acute phase reactions and hematopoiesis, and can play a central role in inflammation of the host defense and tissue injuries [32]. The expression of IL-6 in immunohistochemistry was analyzed after treatment with pioglitazone and LPSF/GQ-02. The control group exhibited a weak reactivity to IL-6 in both the 15-day and 30-day groups (Figs 4A and 5A). The use of a high-fat diet significantly increased the expression of this cytokine in the hepatic tissue, exhibiting immunoreactivity in the cytoplasm of the Kupffer cells and in the membranes of the hepatocytes (Figs 4B and 5B). After the administration of pioglitazone, a staining pattern similar to the HFD group was observed, indicating that the drug was not effective in reducing the expression of this inflammatory cytokine (Figs 4C and 5C). However, in both the 15 and 30-day groups, LPSF/GQ-02 significantly decreased the expression of IL-6 when compared to the HFD and pioglitazone groups, exhibiting a labelling pattern similar to the control (Figs 4D and 5D).

After analyzing the expression of iNOS in the hepatic tissue, the 15 and 30-day control groups exhibited a low reactivity to iNOS (Figs 4E and 5E). The HFD group significantly increased reactivity to iNOS, with diffuse marking in the cytoplasm of the hepatocytes of animals treated for 15 and 30 days, indicating an increase in the inflammatory process (Figs 4F and 5F). The groups treated with pioglitazone for 15 and 30 days exhibited a strong reactivity to iNOS, similar to the HFD group, indicating an intense labelling throughout the cytoplasm of the hepatocytes, as well as in the Kupffer cells, suggesting that pioglitazone increases the inflammatory process in the hepatic tissue (Figs 4 and 5G). After the administration of LPSF/GQ-02 for 15 days, there was a significant decrease in the staining of iNOS in all of the hepatic tissue, when compared with the HFD and pioglitazone groups (Fig 4H). The group treated with LPSF/GQ-02 for 30 days did not exhibit any specific staining for iNOS (Fig 5H), suggesting that LPSF/GQ-02 participates in the inflammatory process in a beneficial manner, decreasing the expression of iNOS and contributing to an improvement in the hepatic tissue.

In an attempt to elucidate the anti-inflammatory mechanisms of LPSF/GQ-02, the expression of COX-2 was assessed through immunohistochemistry after treatment with pioglitazone and LPSF/GQ-02 in LDLR-/- animals submitted to a high-fat diet. Both the 15-day and 30-day control groups exhibited low reactivity to the enzyme COX-2 (Figs 4I and 5I). After exposure to a high-fat diet for 15 or 30 days, a strong immunoreactivity to the enzyme COX-2 was recorded, especially in the cytoplasm of hepatocytes (Figs 4J and 5J), which indicates that the high-fat diet contributed to the hepatic inflammation process. In the pioglitazone group, reactivity to COX-2 was similar to the HFD group, indicating that pioglitazone does not help to reduce inflammation in this animal model (Figs 4K and 5K). However, after the use of LPSF/GQ-02, a significant reduction in the immunoreaction for COX-2 was observed when compared to the HFD and pioglitazone groups, both for 15 and 30 days (Figs 4L and 5L). These findings strengthen the theory that the thiazolidine derivative LPSF/GQ-02 helps to reduce the inflammatory process.

Since the involvement of Kupffer cells is important in the hepatic inflammatory process, F4/80, a specific marker for Kupffer cells, was used to assess their expression in hepatic tissue after treatment with LPSF/GQ-02. Both the 15-day and 30-day control groups exhibited low positivity to F4/80 (Figs 4M and 5M) in all of the hepatic tissue. After the administration of the diet, increased immunoreactivity to F4/80 was recorded, especially in the 15-day group, indicating an increase in the population of Kupffer cells in the hepatic tissue (Figs 4N and 5N). The administration of pioglitazone led to a reduction in the positivity of cells to F4/80, especially in the 15-day group, although this reactivity was not significant in either group, indicating the presence of Kupffer cells and possibly an inflammatory process (Figs 4O and 5O). LPSF/GQ-02 exhibited less immunoreactivity for F4/80. The 15-day and 30-day groups exhibited low reactivity in all of the hepatic tissue, with a significant decrease when compared with the HFD and pioglitazone groups (Figs 4P and 5P), which corroborates previous results, indicating that LPSF/GQ-02 decreases hepatic inflammation

All of the quantitative analysis was performed using Gimp 2.6 software.

### Effects of LPSF/GQ-02 on the Protein Expression of PPARα, PPARγ, IκBα, NFκB p65, eNOS, CD-220, IRS-1 and ABCA1

**Fig 6:**
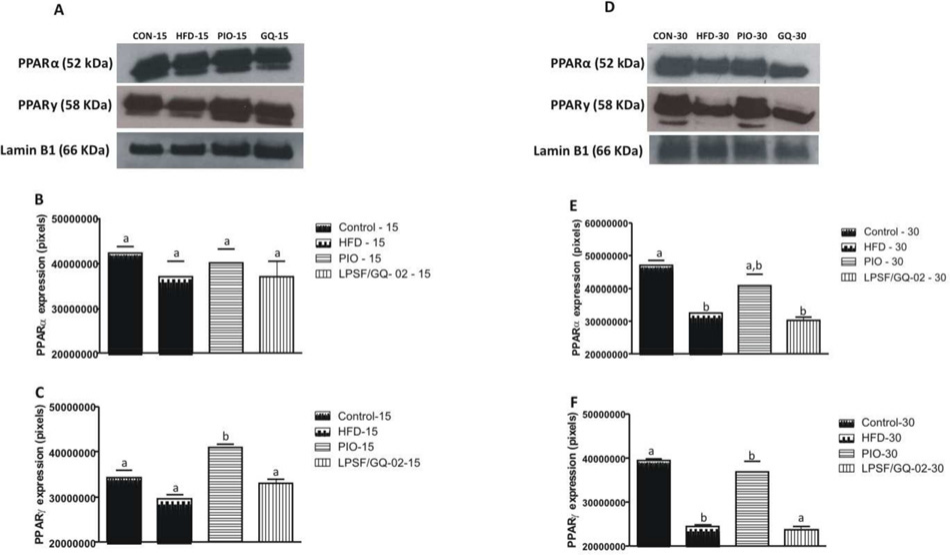
Western blotting analysis showing the effects of Pioglitazone and LPSF/GQ-02 on the expression of PPARα and PPARγ in the liver of LDLR-/- mice. Treatment for 15 days did not alter the expression of PPARα (A, B). However, in the 30-day experiment, the HFD and LPSF/GQ-02 groups exhibited a decrease in the expression of PPARα when compared to the control groups (D, E). Treatment for 15 days only increased the expression of PPARγ in the pioglitazone group in relation to all other groups. (A, C) In the 30-day experiment, the HFD and LPSF/GQ-02 groups exhibited a decrease in the expression of PPARγ when compared to the control and pioglitazone groups. (D, F) The data were analyzed using the Student’s t-test. The columns represent the mean ± S.D. of the protein investigated. The results were confirmed in three different experiments (n = 5). Different letters denote significant differences between treatments, P< 0.05.

The protein levels of nuclear PPARα were assessed after treatment with pioglitazone and LPSF/GQ-02. No significant differences were found in the PPARα protein levels in the groups treated for 15 days (Fig 6A and 6B). However, in the 30-day experiment, the HFD group exhibited a significant reduction in the expression of PPARα when compared to the control group (Fig 6D and 6E). In addition, treatment with LPSF/GQ-02 was not capable of increasing the expression of PPARα, similar to the HFD group (Fig 6D and 6E).

After assessing the protein expression of nuclear PPARγ, it is notable that among the animals treated for 15 days, only the pioglitazone group exhibited a significant difference in relation to all of the other groups (Fig 6A and 6C), thereby confirming its action as an agonist of PPARγ. Meanwhile, the animals that were treated for 30 days with pioglitazone and LPSF/GQ-02 exhibited the following results: the HFD group significantly decreased the levels of PPARγ when compared to the control and pioglitazone groups, with no alteration in relation to the LPSF/GQ-02 group. Furthermore, the LPSF/GQ-02 group exhibited levels of expression of PPARγ that were similar to the HFD group (Fig 6D and 6F).

**Fig 7:**
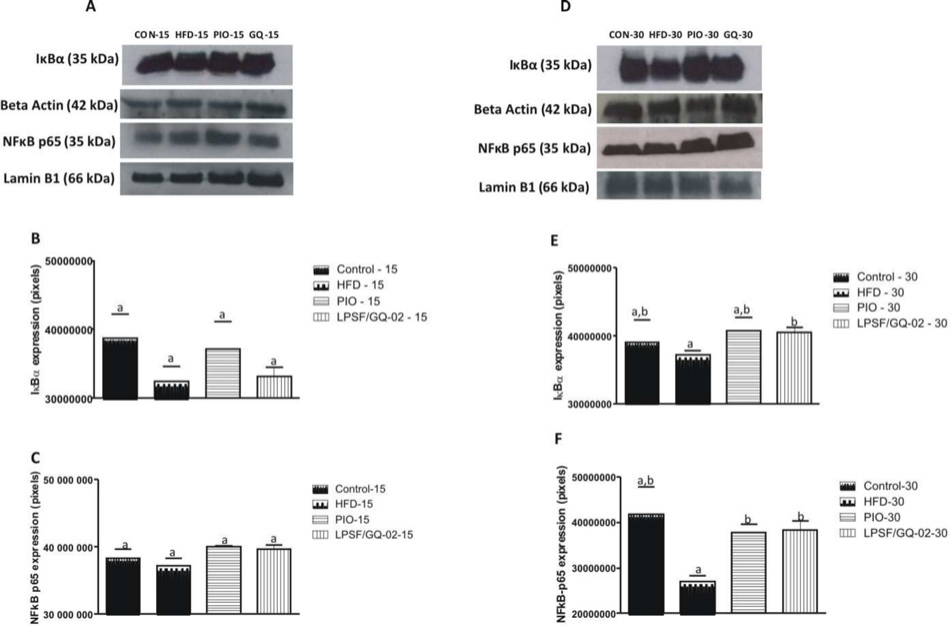
Western blotting analysis showing the effects of Pioglitazone and LPSF/GQ-02 on the expression of IkBα and NFkB p65 in the liver of LDLR-/- mice. Treatment for 15 days did not alter the expression of the protein IkBα (A, B). However, the prolonged treatment with LPSF/GQ-02 significantly increased the expression of IkBα when compared to the control group (D, E). Treatment for 15 days did not alter the expression of the protein NFkB p65 (A, C) However, the prolonged treatment with pioglitazone and LPSF/GQ-02 significantly increased the expression of NFkB p65 when compared to the HFD group (D, F). The data was analyzed using the Student’s t-test. The columns represent the mean ± S.D. of the protein investigated. The results were confirmed in three different experiments (n = 5). Different letters denote significant differences between treatments, P< 0.05.

Expression of the protein IκBα was analyzed through western blotting on the hepatic tissue after treatment with pioglitazone and LPSF/GQ-02. As a result, there was no alteration in the levels of this protein after treatment with pioglitazone and LPSF/GQ-02 for 15 days (Fig 7A and 7B). However, the prolonged treatment (30 days) led to a significant increase of the protein IκBα in the group treated with LPSF/GQ-02 when compared to the HFD group (Fig 7D and 7E).

It is known that NFκB is involved in the hepatic inflammatory process, activating different cytokines, and consequently, the present study assessed the cytosolic levels of this transcription factor in the hepatic tissue after the administration of pioglitazone and LPSF/GQ-02. The levels of NFκB-p65 in the animals that were treated for 15 days did not alter after the administration of the drug (Fig 7A and 7C). However, after using the drug for 30 days, it was possible to observe a significant increase in the expression of cytoplasmic NFκB-65 in the pioglitazone and LPSF/GQ-02 groups when compared with the HFD group (Fig 7D and 7F).

**Fig 8:**
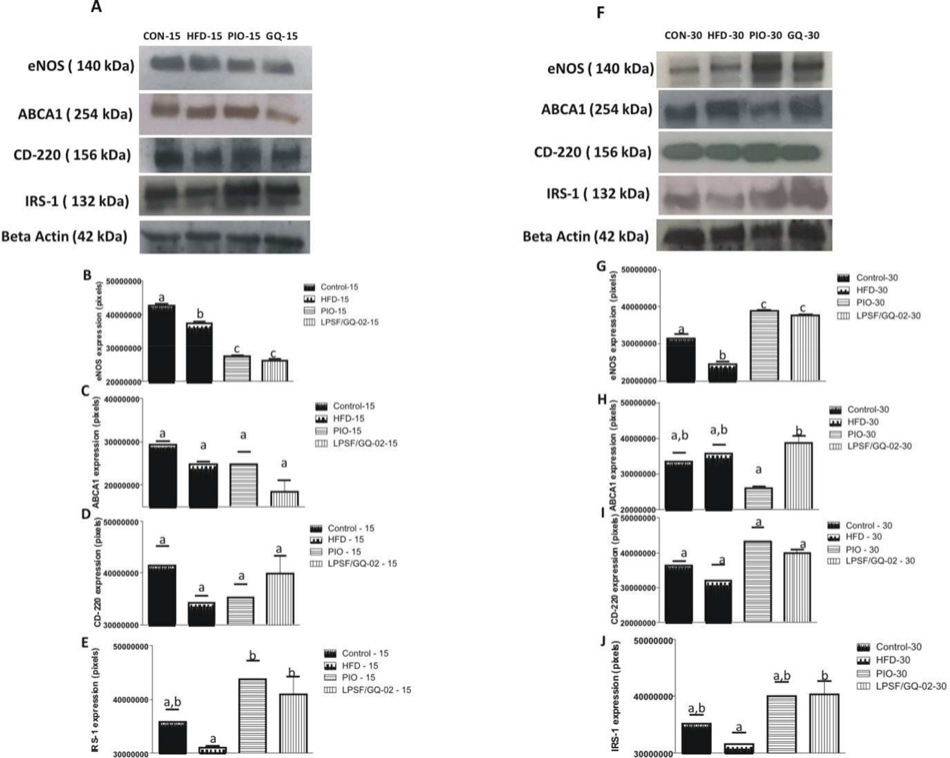
Western blotting analysis showing the effects of Pioglitazone and LPSF/GQ-02 on the expression of eNOS, ABCA1, CD-220 and IRS-1 in the liver of LDLR-/- mice. Treatment for 15 days reduced the concentration of eNOS in the pioglitazone and LPSF/GQ-02 groups in comparison with the control and HFD groups (A,B) However, after 30 days, an increase was observed in the expression of eNOS in the pioglitazone and LPSF/GQ-02 groups in comparison to the HFD group (F,G). The 15-day treatment did not alter the levels of ABCA1 in relation to all of the groups. (A,C) After 30 days, significant differences of ABCA1 were only found between the pioglitazone and LPSF/GQ-02 groups. (F,H). Treatment for 15 or 30 days did not alter the expression of the insulin receptor CD-220 in all of the groups studied (A,D,F,I). The 15-day treatment with pioglitazone and LPSF/GQ-02 increased the protein levels of IRS-1 when compared with the HFD group. (A,E) LPSF/GQ-02 increased the levels of IRS-1 after thirty days in comparison with the HFD group. (F,J). The data was analyzed using the Student’s t-test. The columns represent the mean ± S.D. of the protein investigated. The results were confirmed in three different experiments (n = 5). Different letters denote significant differences between treatments, P< 0.05.

The integrity of the hepatic sinusoidal endothelium is extremely important for the maintenance of hepatic physiology and disturbance of the sinusoidal endothelium function could play an important role in the physiopathology of the liver. The expression of eNOS was assessed by western blotting after treatment with pioglitazone and LPSF/GQ-02. The HFD, pioglitazone and LPSF/GQ-02 groups exhibited a significant reduction in the expression of eNOS after 15 days of treatment (Fig 8A and 8B). However, after using the drugs for 30 days, a significant increase in the expression of eNOS was observed when compared to the HFD group (Fig 8F and 8G), indicating that the increase in eNOS expression was time-dependant, after the administration of the drugs.

Insulin resistance is directly associated with the development of NAFLD. Therefore, markers of the insulin signaling pathway were assessed after treatment with pioglitazone and LPSF/GQ-02. Although the protein levels of the insulin receptor (CD-220) increased after treatment with pioglitazone and LPSF/GQ-02, this increase was not significant after either 15 or 30 days of treatment and there was no significant difference between the groups (Fig 8A and 8D/ Fig 8F and 8I). However, after treatment with pioglitazone and LPSF/GQ-02 for 15 days, there was a significant increase in the protein levels of the insulin receptor substrate (IRS-1) when compared to the HFD group (Fig 8A and 8E). Furthermore, after 30 days administering these drugs, only the LPSF/GQ-02 group induced a significant increase in the levels of IRS-1 when compared with the HFD (Fig 8F and 8J), indicating an improvement in the signaling cascade of insulin.

ATP-binding cassette transporter AI (ABCA1) is a member of the ABC family of transporters that are necessary for the formation of HDL plasma. After treatment for 15 days, no significant differences were found in the protein expression of ABCA1 in relation to all of the groups (Fig 8A and 8C). Similarly, after treatment for 30 days, although there was an increase in the expression of ABCA1 in the LPSF/GQ-02 group, this increase was only significant when compared to the pioglitazone group (Fig 8F and 8H)

## Discussion

Disturbances in the lipid profile, such as an increase in TGs and LDL and a reduction in HDL, are associated with NAFLD [33]. In the present study, Western diet induced significant increases in the TGs, TC and LDL profiles, similar results were achieved after treatment with pioglitazone and LPSF/GQ-02, although pioglitazone induced higher levels of LDL when compared to the HFD group. These results are according with the fact that PPARγ agonists (TZDs) do not have favorable effects on the lipid profile and are not considered a lipid-lowering class of drugs [34, 35]. However, after 30 days of treatment with pioglitazone and LPSF/GQ-02 improved plasmatic HDL was observed, possibly by promoting cholesterol efflux PPARγ dependent mechanism [36].

TZDs are considered blood glucose-lowering drugs [37] by increasing the uptake of insulin-mediated glucose in skeletal muscle, suppress the production of hepatic glucose, and improve the secretion of insulin in β cells of the pancreas [38]. In the present study, the animals treated with pioglitazone and LPSF/GQ-02 promoted an accentuated decrease in glucose levels in comparison to the HFD group. These results confirmed previous studies that demonstrated a beneficial effect on insulin sensibility of LPSF/GQ-02 [24].

According to Yang et al. [39] the main histological characteristic of NAFLD is excessive accumulation of triglycerides in hepatocytes. In addition, obesity and insulin resistance lead to an increase in the hepatic flow of free fatty acids and the accumulation of fat in the liver [40]. In the present study, histological sections were used to analyze the hepatic architecture after the administration of pioglitazone and LPSF/GQ-02. The results showed an improvement in all hepatic tissue after the use of LPSF/GQ-02, for 15 or 30 days, as evidenced by decreased vacuolization and inflammatory infiltrates, as well as better organization in all of the hepatic parenchyma. Controversially, the group that received pioglitazone was not effective in reducing hepatic damage, exhibiting similar characteristics to the HFD group. Pioglitazone is a drug classified as thiazolidinedione and is considered an agonist of PPARγ. It is known that PPARγ is elevated in murine models of diabetes and obesity [41–43]. Furthermore, the deletion of PPARγ from hepatocytes protects mice against hepatic steatosis induced by a high-fat diet [44]. These results suggest a strong association between the accumulation of fat in the liver and elevated levels of PPARγ. Recently, Zhang, et al. [45] observed that the administration of pioglitazone decreased hepatic steatosis in rats with NASH, induced by a high-fat diet. Based on all of these findings, it remains controversial to confirm if PPARγ is the causal factor or a consequence of the accumulation of fat in the liver.

Oil red O, a specific coloration for lipids, and the hepatic triglyceride dosage were used to confirm the reduction of fat in the liver of animals treated with LPSF/GQ-02. As expected, there was a significant reduction in the concentration of lipids in the livers of animals treated with LPSF/GQ-02 in both the 15-day and 30-day groups, indicating that the thiazolidine derivative LPSF/GQ-02 was effective in reducing fat in the livers of animals fed with a high-fat diet. On the other hand, the hepatic tissue of the animals treated with pioglitazone maintained a large accumulation of lipids, confirming the results found in the histology, in which pioglitazone was not effective in decreasing hepatic steatosis. Controversially, Wang et al. [46] showed that rosiglitazone reduced the levels of hepatic lipids evidenced by the reduction in Oil red O staining in rats fed with a high-fat diet. It is possible that the damaging effects exhibited by pioglitazone in the present study are specific to this molecule and do not represent a class effect. However, the determining mechanisms for the increase in hepatic steatosis associated with TZDs remain unknown.

According to Ciupin´ska-Kajor et al [47], the fibrosis process in the liver is considered the most important step in the progression of NAFLD. Hepatic fibrosis involves the disorganization of the architecture of the hepatic tissue and the accumulation of extracellular matrix in response to pathological insults [48]. For this reason, the quantity of collagen was assessed by means of specific Sirius red staining. Similar to the results found in the histology and the staining of lipids, both the 15-day and 30-day treatment groups with LPSF/GQ-02 significantly decreased the quantity of collagen in comparison to the HFD group. On the other hand, the pioglitazone group exhibited a time-dependant response, with a significant reduction of collagen only found in the 30-day group. These results suggest effective action by LPSF/GQ-02 on the morphological factors that endanger normal liver function, leading to the development of NAFLD.

In the inflammatory profile, IL—6 is a key element in the acute phase response, mediating the synthesis of different proteins of the acute phase (such as protein C—reactive and serum amyloid A) [49]. Furthermore, IL—6 is considered as a marker of the prognostic of resistance to insulin and cardiovascular diseases. Elevated serum levels of IL-6 are present in animal models and in patients with NAFLD [50–52]. Similarly, Mas et al [53] demonstrated that diet-induced NASH was reduced in IL-6 knockout mice, indicating that this cytokine has a pro-inflammatory action during hepatic diseases. In the present study, LPSF/GQ-02 significantly reduced the expression of IL-6, indicating a possible hepatic anti-inflammatory action. Controversially, in the present study, pioglitazone increased the levels of IL-6 after 15 and 30 days of treatment. Esterson et al [54] studied biopsies of the adipose tissue of non-obese and obese individuals with type 2 diabetes and reported that pioglitazone was capable of reducing the levels of IL-6 in obese individuals, although it was not effective in reducing the levels of this cytokine in non-obese individuals. Mohapatra et al. [55] studied female db/db mice and concluded that pioglitazone was effective in reducing the plasma levels of IL-6 in mice that received a sub-therapeutic (3 mg/kg) or therapeutic dose of pioglitazone (30 mg/kg). These different results related to the effect of pioglitazone on the levels of IL-6 could be partly due to the experimental model used.

During inflammation, inducible nitric oxide synthase (iNOS) plays an important role in the exacerbated production of nitric oxide, contributing to tissue damage [56]. Nitric oxide (NO) is produced endogenously by iNOS and leads to the formation of reactive oxygen species (ROS) and reactive nitrogen species (RNS). NO can react rapidly with superoxide anion and produce peroxynitrite, leading to the nitration of proteins [57]. In the present study, the expression of iNOS was significantly reduced after using LPSF/GQ-02 in LDLR-/- mice submitted to a HFD. Similarly, Salamone and collaborators administered silibinin and reported a significant reduction in the expression of iNOS in db/db mice submitted to experimental non-alcoholic steatohepatitis [57].

Cicloxigenase-2 (COX-2) is a key enzyme in the activation of the inflammatory response. Inflammation caused by the activation of COX-2 in the hepatic tissue plays an important role in the development of insulin resistance and NAFLD [58]. According to Yu et al. [59] the use of COX-2 inhibitors could protect against NASH in animal models. In the present study, LPSF/GQ-02 effectively reduced the expression of COX-2 in an animal model of NAFLD, strengthening the hypothesis that this thiazolidine derivative decreases inflammation in the hepatic tissue. Furthermore, these results corroborate the findings of Silva et al. [24], who reported that LPSF/GQ-02 was effective in reducing insulin resistance.

Kupffer cells mediate the hepatic response in relation to the numerous inflammatory stimuli and can play an important role in the progression from steatosis to NAFLD [60]. In a model of NASH induced by a high-fat diet, Kupffer cells were widely recruited and activated [61]. Rivera et al. [62] used a murine model of NASH and confirmed that the reduction in the number of Kupffer cells can attenuate the histological appearance of hepatic steatosis, inflammation and necrosis, suggesting that this cellular type contributes to the pathogenesis of NASH/NAFLD. For this reason, the expression of F4/80 in the hepatic tissue was assessed after treatment with LPSF/GQ-02. The thiazolidine derivative LPSF/GQ-02 was effective in reducing positive marking for F4/80 in comparison to the HFD group, indicating the presence of a smaller population of Kupffer cells n the hepatic tissue. On the other hand, pioglitazone did not reduce the positive marking for macrophages, as evidenced by the great reactivity of F4/80 in the hepatic tissue. Similar to the results found with LPSF/GQ-02, the use of new agonists to PPARα, such as Wy 14643, was effective in reducing the recruitment of macrophages marked by F4/80, as evidenced by the immunohistochemistry of female foz/foz mice [63]. These results suggest that LPSF/GQ-02 acts directly on the inflammation, reducing the recruitment of Kupffer cells and consequently improving the pathological process triggered by NAFLD.

Interestingly, in the present study, pioglitazone did not exhibit hepatic anti-inflammatory effects when the markers IL-6, COX-2, iNOS and F4/80 were assessed. The anti-inflammatory activity of LPSF/GQ-02 may be due to a possible action in other molecular targets.

In an attempt to understand the possible active mechanism of LPSF/GQ-02 in the improvement of hepatic pathological processes, the expression of IκBα, PPARα, PPARγ, IRS-1, CD-220, ABCA1, eNOS and NFκB p65 was analyzed by western blotting analysis.

PPARα is expressed metabolically in active tissues such as the heart, kidneys, intestinal mucous, skeletal mucous and liver, regulating genes involved in the lipid metabolism, gluconeogenesis and amino acids [64]. As well as its effects on the lipid metabolism, PPARα also acts on pro-inflammatory pathways and negatively affects other signaling pathways, such as NFκB. As a result of its effects on the lipid metabolism and inflammation, PPARα can modulate physiopathological mechanisms implicated in NAFLD and atherosclerosis [65]. After assessing the expression of PPARα in the hepatic tissue, no significant alterations were found in the groups treated with LPSF/GQ-02, suggesting that this thiazolidine derivative does not affect the expression of PPARα. The reduction of lipid content, as well as the reduction of inflammation after treatment with LPSF/GQ-02, could be explained by a possible PPARα agonist action. However, this was not confirmed in the results obtained in the present study.

PPARγ is expressed in elevated levels in adipose tissue and plays an important role in the increased sensitivity to insulin, as well as promoting the capture of fatty acids for adipocytes and their differentiation. The consequent effect of these processes results in increased storage of triglycerides in the adipocytes, reducing the delivery of fatty acids to the liver [66]. On the other hand, it is widely known that the increased expression of PPARγ in the hepatic tissue in a murine model plays an important deleterious role, increasing hepatic steatosis [67]. According to Costa-Leite et al. [25], the molecular structure of LPSF/GQ-02 indicates a possible role as an agonist of PPARγ. For this reason, the expression of PPARγ was analyzed after treatment with pioglitazone and LPSF/GQ-02. Treatment with pioglitazone increased the expression of PPARγ, although LPSF/GQ-02 was not capable of inducing significant alterations. These results confirm the agonist action of pioglitazone in relation to PPARγ, as previously described [27, 34].

The κB transcription factor (NFκB) is located (although inactive) in the cellular cytoplasm associated with regulatory proteins called κB inhibitors (IκB), such as IκBα [68]. The phosphorylation of IκBα is an important step in the activation of NFκB and, thus, these proteins become targets for specific therapy, given that the activation of NFκB is associated with the transcription of pro-inflammatory genes [69]. In the present study, prolonged treatment with LPSF/GQ-02 for 30 days induced a significant increase in the expression of IκBα. In addition, there was a significant increase in the total expression of cytoplasmic NFκB-65 in the LPSF/GQ-02 group, when compared with the HFD group, indicating an anti-inflammatory action through the inhibition of NFκB-65. By activating NFκB, IκBα liberates NFκB, thereby enabling the NFκB to be translocated to the nucleus [70]. Therefore, it is possible to infer indirectly that the greater the expression of IκBα and NFκB-65 in the cytoplasm, the lower the translocation to the nucleus will be, with a consequent decrease in inflammatory gene transcription. These results confirm the reduction of hepatic inflammation in the group treated with LPSF/GQ-02, as evidenced by the decrease in the tissue levels of IL-6, iNOS, COX-2 and F4/80.

Endothelial sinusoidal dysfunction, with the decrease in the production of intra-hepatic nitric oxide, has been considered for many years as a relevant pathogenic factor in the progression of hepatic deleterious events, such as cirrhosis [71]. Adequately functioning endothelial sinusoidal cells produce NO, inhibiting the activation of hepatic stellate cells (HSC) and thus, representing a powerful natural antifibrotic [72]. Moreover, NO produced by the enzyme endothelial nitric oxide synthase (eNOS), in nanomolar concentrations, exhibits anti-inflammatory effects through the signaling pathways of the GMPc and can inhibit the activity of NFκB, among other things [73,74]. After the administration of LPSF/GQ-02, a significant increase of eNOS was observed in comparison to the control group, indicating that LPSF/GQ-02 could develop its anti-inflammatory and antifibrotic activities partly due to the activation of this enzyme in the hepatic tissue. It is interesting to note that the treatment with LPSF/GQ-02 was effective in decreasing the quantity of hepatic collagen and thereby reducing tissue fibrosis, as evidenced by the results obtained with eNOS.

Hepatic resistance to insulin is associated with NAFLD and is an important factor in the pathogenesis of type 2 diabetes and metabolic syndrome [75]. Although there is a general consensus that insulin resistance is caused by defects in insulin signaling, many causes have been proposed to explain how these insulin signaling defects appear in NAFLD. Inflammation, oxidative stress of the endoplasmic reticulum and the accumulation of lipids in the liver have been indicated as causes for the development of insulin resistance in animal models of NAFLD [76]. Furthermore, the production of cytokines is known to activate intracellular kinases capable of inhibiting key elements of the insulin signaling route, including proteins of the insulin receptor substrate (IRS) and phosphatidylinositol 3-kinase (PI3K) [77]. For this reason, the expression of two important molecules of the insulin signaling route was analyzed after the administration of LPSF/GQ-02: insulin receptor (CD-220) and IRS-1. Although there were no alterations in the levels of the insulin receptor, LPSF/GQ-02 significantly increased the levels of IRS-1 in comparison to the HFD group. These results possibly indicates an improving of the insulin signaling, although it is necessary to be confirmed by analyzing Serine/Threonine or Tyrosine phosphorylation grade of IRS-1, akt/PKB or GSK, since they reflect the real state of the hepatic insulin signaling. In a previous study conducted in our laboratory, SILVA et al. [24] observed that LPSF/GQ-02 was capable of improving the sensitivity to insulin in LDLR-/- mice fed with a high-fat diet. Thus, the accumulation of hepatic fat and inflammation seem to be partly associated with altered insulin signaling in animals fed with a high-fat diet, which is restored after the treatment with LPSF/GQ-02.

ATP-binding cassette transporter AI (ABCA1) is a part of the ABC family of transporters. The fundamental role of ABCA1 in the formation of foam cells and atherosclerosis has already been established: it mediates the active transport of intracellular cholesterol and phospholipids to apolipoprotein AI, which is the main lipoprotein of the HDL. Mutations of the ABCA1 gene cause a decrease in the levels of HDL cholesterol, with a consequent increase in atherosclerosis [78]. Although not yet fully defined, it is known that the expression of apolipoprotein AI or ABCA1 in hepatocytes can reduce hepatic steatosis, decreasing the storage of lipids in the hepatocytes by transporting the lipids and also by reducing the oxidative stress of the endoplasmic reticulum, which further favors the diminishment of steatosis [79]. In the present study, although there was an increase in the protein levels of ABCA1 in the hepatic tissue after treatment with LPSF/GQ-02, this increase was not significant in relation to the HFD group. According to Ozasa et al [36], pioglitazone was effective in the efflux of cholesterol, based on the expression of ABCA1, in a manner that was dependant on PPARγ in atherosclerotic plaque. Contradictorily, in the present study, the pioglitazone group also did not increase the protein levels of ABCA1 after the use of the high-fat diet. These differences may be due to the different cellular types studied.

In summary, the present study demonstrated that the chronic consumption of a high-fat diet developed NAFLD characteristics in LDLR-/- mice while also increasing fat, hepatic fibrosis, inflammation and insulin resistance. The administration of LPSF/GQ-02 inhibited the hepatic injury, decreased inflammatory markers and increased sensitivity to insulin, suggesting an important role in the improvement of NAFLD. Therefore, LPSF/GQ-02 has become a potential candidate for the treatment of chronic hepatic pathologies triggered by the consumption of a high-fat diet. New studies are being conducted in our laboratory to investigate the possible active mechanisms of LPSF/GQ-02 on the hepatic lipid metabolism.
